# Exploring the immunometabolic potential of Danggui Buxue Decoction for the treatment of IBD-related colorectal cancer

**DOI:** 10.1186/s13020-024-00978-y

**Published:** 2024-08-29

**Authors:** Yang Zhang, Qianming Kang, Luying He, Ka Iong Chan, Hui Gu, Wenjing Xue, Zhangfeng Zhong, Wen Tan

**Affiliations:** 1 School of Pharmacy, Lanzhou University, Lanzhou, 730000 China; 2 Macao Centre for Research and Development in Chinese Medicine, Institute of Chinese Medical Sciences, University of Macau, Macao, 999078 SAR China

**Keywords:** Danggui Buxue Decoction, Astragali Radix, Angelicae Sinensis Radix, Immunometabolism, IBD-CRC

## Abstract

Danggui Buxue (DGBX) decoction is a classical prescription composed of Astragali Radix (AR) and Angelicae Sinensis Radix (ASR), used to enrich blood, and nourish Qi in Chinese medicine, with the potential to recover energy and stimulate metabolism. Chronic inflammation is a risk factor in the development of inflammatory bowel disease (IBD)-related colorectal cancer (CRC). More importantly, AR and ASR have anti-inflammatory and anti-cancer activities, as well as prefiguring a potential effect on inflammation-cancer transformation. We, therefore, aimed to review the immunometabolism potential of DGBX decoction and its components in this malignant transformation, to provide a helpful complement to manage the risk of IBD-CRC. The present study investigates the multifaceted roles of DGBX decoction and its entire components AR and ASR, including anti-inflammation effects, anti-cancer properties, immune regulation, and metabolic regulation. This assessment is informed by a synthesis of scholarly literature, with more than two hundred articles retrieved from PubMed, Web of Science, and Scopus databases within the past two decades. The search strategy employed utilized keywords such as “Danggui Buxue”, “Astragali Radix”, “Angelicae Sinensis Radix”, “Inflammation”, and “Metabolism”, alongside the related synonyms, with a particular emphasis on high-quality research and studies yielding significant findings. The potential of DGBX decoction in modulating immunometabolism holds promise for the treatment of IBD-related CRC. It is particularly relevant given the heterogeneity of CRC and the growing trend towards personalized medicine, but the precise and detailed mechanism necessitate further in vivo validation and extensive clinical studies to substantiate the immunometabolic modulation and delineate the pathways involved.

**Graphical Abstract:**

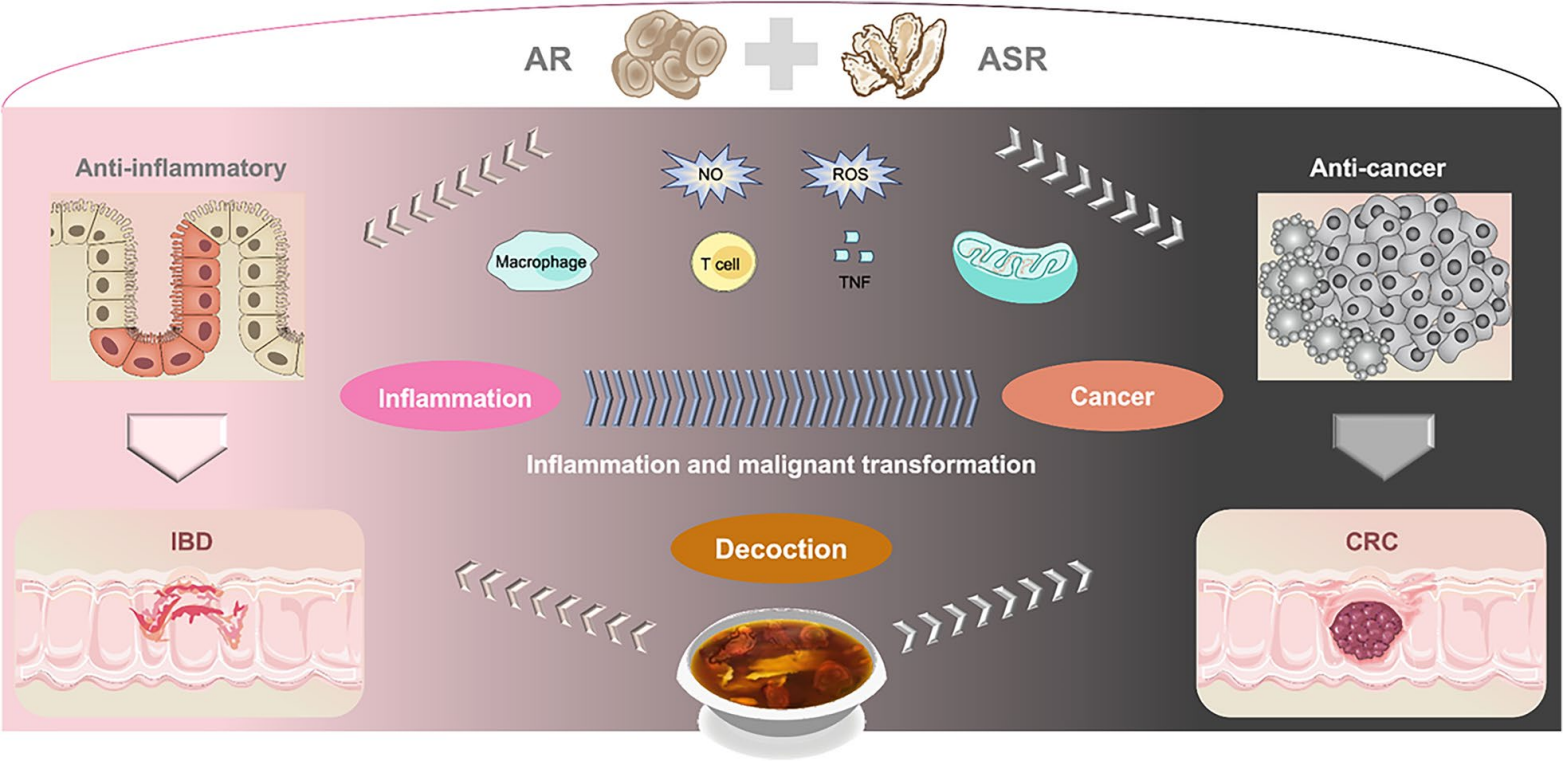

## Highlights

- Danggui Buxue decoction is a classical prescription consisting of Astragali Radix and Angelicae Sinensis Radix with the efficacy of tonifying blood and invigorating qi in traditional Chinese medicine.
- Astragali Radix and Angelicae Sinensis Radix, both have a variety of pharmacological activities, including anti-inflammation and anti-cancer effects.
- In view of inflammation and malignant transformation in inflammatory bowel disease-related colorectal cancer and the curative effects of Astragali Radix and Angelicae Sinensis Radix, immunometabolism modulation potential of DGBX were reviewed and discussed in the present study.

Danggui Buxue (DGBX) decoction is a classical prescription consisting of Astragali Radix (AR) and Angelicae Sinensis Radix (ASR) in traditional Chinese medicine. Given the documented anti-inflammatory and anti-neoplastic properties, this review aims to discuss the potential of mitigating the inflammation-cancer transformation and to offer an immunometabolic adjunct in IBD-CRC risk management.

## Overview of IBD and CRC

### Pathogenesis and epidemiological characteristics of IBD

IBD includes ulcerative colitis (UC) and Crohn’s disease (CD) and is a chronic inflammatory disease occurring in the gastrointestinal tract [1]. UC characteristically initiates in the rectum, and subsequently spreads to the entire colon in a continuous manner, while CD predominately involves the terminal ileum and perianal region with a discontinuous pattern of involvement extending throughout the gastrointestinal tract [2]. UC inflammation in the mucous membrane leads to ulcers and bloody diarrhea [3]. CD typically involves abdominal pain, chronic diarrhea, weight of loss, and fatigue [4]. In the past, IBD was regarded as a Western disease; however, in the twenty-first century, the incidence and prevalence of IBD are increasing worldwide. Although still lower than in Western countries, the incidence and prevalence of IBD in Asia is increasing over time [5]. Therefore, comprehending the evolving epidemiological patterns and pathogenesis thereof is crucial in addressing the escalating global burden. The pathogenesis of IBD is related to heredity, the intestinal microbe, the environment, and immunity [6]. Genome-wide associated studies of genes and genetic loci involved in IBD identified 99 non-overlapping genetic risk loci and revealed the exact role of disease-related genes. Nucleotide-binding oligomerization domain containing protein 2 (NOD2), for example, is appropriately regulated to maintain intestinal homeostasis [2]. Tens of thousands of microbes living in the human gut are involved in the regulation of health and disease [7], and the human gut contains more than 2000 species of microbes, including Firmicutes, Bacteroides, Actinomycetes, and Proteus [8]. Patients with IBD have significantly less microbial diversity than that of healthy individuals [9]. Environmental determinants, such as tobacco smoking, appendectomy, oral contraceptive use, and dietary habits, exert distinct influences on the risk profiles for celiac disease and CD. Appendectomy has different effects on UC and CD, with a general protective effect and reduced risk of UC, and with an increased risk of stenosis and reduced risk of anal fistula in CD [10, 11]. Contraceptive use in women with a history of smoking is also associated with the occurrence and development of IBD [12, 13]. Even though there is no conclusive evidence that dietary factors are directly related to the pathogenesis of IBD, low-fiber and high-fat foods have been proposed as risk factors [14]. In healthy people, the initial immune response is rigorously regulated, this regulation determines immune tolerance or defensive inflammatory responses, and some disturbances in the balance of these responses may lead to IBD [15]. IBD in patients who are failing to achieve effective disease control may ultimately lead to the development of cancer.

### Pathogenesis and epidemiological characteristics of colorectal cancer

CRC is the fourth leading cause of cancer-related deaths and the most common malignancy worldwide [16]. In 2020, there were nearly 4.56 million newly diagnosed cancer cases and 30 million cancer deaths in China [17]. Of the 147,000 people diagnosed with colorectal cancer, approximately 53,000 will eventually die. Despite variations in CRC incidence and mortality by age, ethnicity, and geographic location, a concerning trend of escalating incidence and mortality rates has been observed for CRC [18]. The susceptibility to CRC is influenced by a spectrum of individual-specific factors, encompassing age, lifestyle, and a history of chronic disease. IBD patient are notably at an elevated risk for the development of CRC. Chronic inflammation is postulated to foster aberrant cell proliferation, and prolonged exposure to inflammation can lead to cellular atypia, potentially culminating in the formation of neoplastic lesion [19–21]. The occurrence and development of CRC goes through several stages, including normal mucosal epithelium, abnormal crypt foci, microadenoma, and finally the malignant tumor. The progression from normal mucosal epithelium to abnormal crypts is ordinarily considered to be the onset of dysplasia, and a single dysplasia crypt is considered the first histological manifestation of a tumor [22]. Adenomatous polyps progressing to sporadic CRC typically undergo a protracted period of development, and CRC associated with colitis is believed to evolve through multiple stages of precursor lesions, ranging from inflammation to low-grade dysplasia, high-grade dysplasia, and finally, CRC [23]. CRC is not an abrupt occurrence; hence, timely detection and treatment during its formation can effectively prevent it.

### Risk and epidemiological characteristics of CRC in IBD

Patients with IBD have an elevated risk of developing CRC, and chronic inflammation leads to dysplastic precursor lesions that may appear in multiple regions of the colon through a local carcinization process. Patients with IBD are at 2–6 times higher risk of developing CRC compared to the general population. IBD-related colorectal cancer accounts for approximately 2% of total annual CRC mortality and 10–15% of annual mortality in patients with IBD [24]. The pathogenesis of IBD-related CRC diverges from that of sporadic CRC, typically manifesting through a distinct sequence characterized by chronic inflammation, dysplastic transformation, and eventual carcinomatous progression. Research has demonstrated that intestinal inflammation can lead to the dysregulation of the host's immune response and a disruption in the homeostasis of the intestinal microbiota. The gut microbiota plays a crucial role in maintaining intestinal homeostasis by impeding pathogen colonization and modulating immune cell networks. *Bacteroides fragilis*, *Fusobacterium nucleatum*, and *Porphyromonas gingivalis* are known to be closely related to IBD-CRC [25]. Intestinal microbiota and their metabolites modulate the metabolic pathways of immune cells, thereby ameliorating IBD within the gastrointestinal tract and augmenting the efficacy of CRC immunotherapy [26–29]. Colonoscopy and staging biopsies should be performed in patients with long-term IBD since early detection of dysplasia is critical for the prevention of CRC [30]. A slow transition from IBD to cancer is associated with chronic inflammation, so reducing inflammation caused by colitis is a preventive approach and strategy to decrease risk of IBD-CRC [31–33]. Chemoprophylaxis is also one of the main means of continuous and complete control of inflammation [34, 35]. The risk of IBD patients developing CRC has decreased recently, which may be due to early monitoring and appropriate treatments.

## Immunometabolism regulation in IBD-related CRC

### Immune regulation in the tumor microenvironment (TME)

The TME is a cellular environment in which the tumor exists, and the continuous interaction between tumor cells and the surrounding microenvironment plays a crucial role in the genesis, progression, and metastasis of tumors. This complex microenvironment consists of tumor cells, stromal cells, and extracellular matrix. Stromal cells include immune cells and the cytokines or chemokines secreted by these cells [36, 37]. Immune cells play an important role in tumorigenesis, including innate immune cells, such as natural killer (NK) cells, macrophages, dendritic cells (DCs), myeloid-derived suppressor cells (MDSCs), and adaptive immune cells, such as T cells and B cells [38, 39]. The cytotoxic activity of NK cells is primarily mediated through two well-characterized mechanisms, one is the release of cytotoxic granules containing perforin and granzymes, and the other is the secretion of pro-inflammatory cytokines. NK cells from IBD patients exhibit a diminished production of interferon-gamma (IFN-γ), yet an increased secretion of tumor necrosis factor-alpha (TNF-α) [40]. Elevated levels of TNF-α have been correlated with the presence of aberrant crypt foci within colorectal polyps [41]. The dynamic equilibrium between M1 and M2 macrophage polarization is a critical determinant of the inflammatory microenvironment and has profound implications for tumor and inflammation [42–45]. Clinical observations have highlighted a significant association between the overexpression of M2 macrophages and the progression of CRC [46, 47]. DCs function as specialized antigen presenting cells, whereas MDSCs consist of monocytes and polymorphonuclear immature bone marrow cells. In the CRC microenvironment, MDSCs represent the predominant immunosuppressive cell population within the TME and play a critical role in promoting immune resistance [48–53]. T cells play a pivotal role in orchestrating the immune response against CRC, rendering them one of the most critical components of immune system. Activated CD8^+^ T cells have cytotoxic effects on CRC cells, while activated CD4^+^ T cells can differentiate into subtypes that promote or inhibit tumor growth. Tumor-infiltrating B lymphocytes are considered the main effector cells of the humoral adaptive immune response, and B cells are recognized in the immune system for their ability to produce antibodies and secrete pro-inflammatory and anti-inflammatory cytokines regulating CRC progression [54]. Immune cells play a multifaceted role in the pathogenesis of CRC, influencing the survival, proliferation, and metastatic potential of CRC cells, and actively participating in the regulation of cancer progression. The activation and differentiation of these immune cells are accompanied by significant metabolic reprogramming, which is essential for their functional capabilities. The unique metabolic characteristics of immune cells also have a profound impact on their ability to perform their immune functions [38, 55].

### Immunometabolism aspects in the TME

**Fig. 1:**
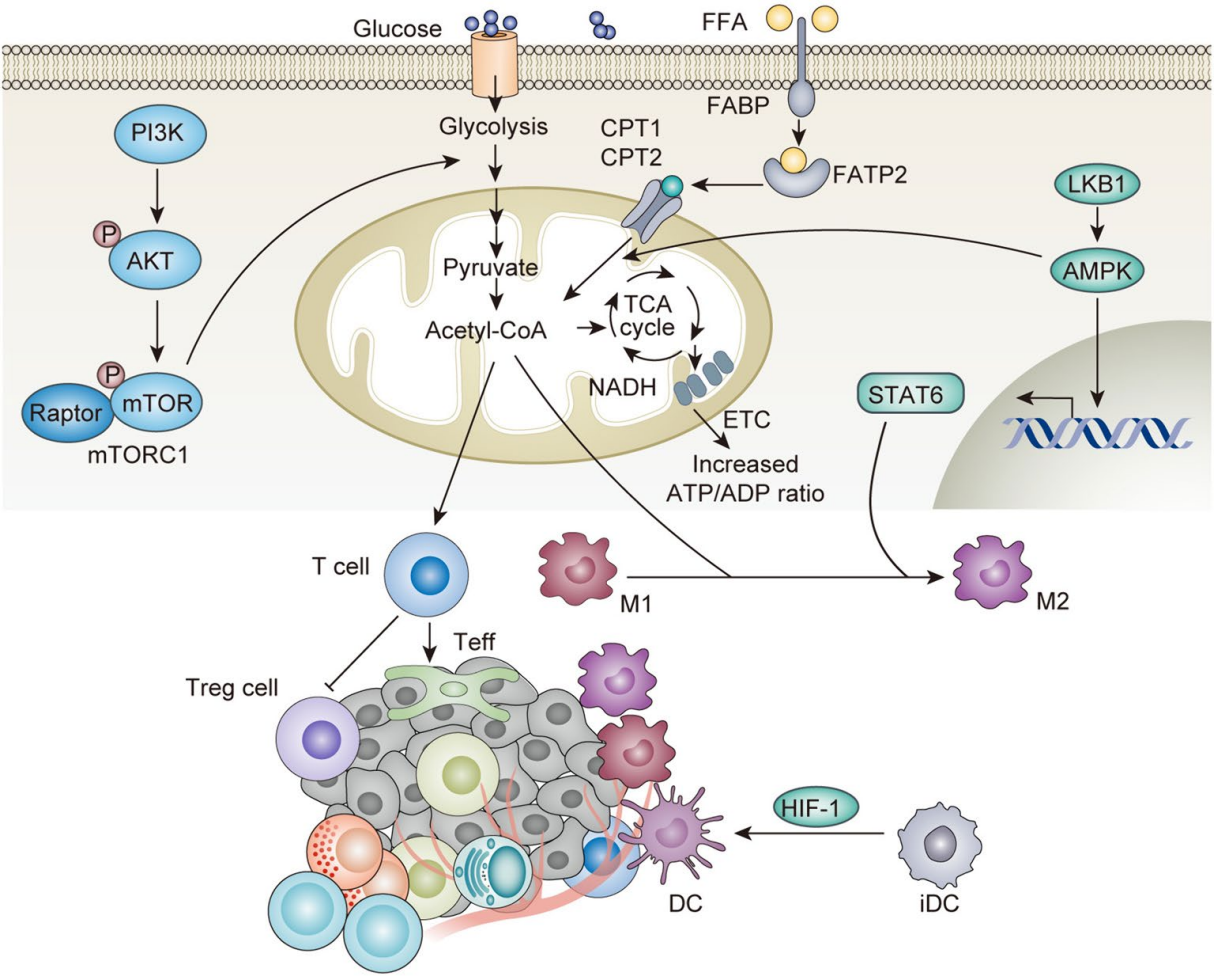
The immunometabolism modulation. LKB1-AMPK signaling pathway and PI3K/AKT/mTOR signaling pathway are the main pathways regulating the metabolism of fatty acids and glucose. Consequently, the metabolic outcomes impact immune cells such as T cells, DCs, M1 and M2 macrophages, thereby influencing immunity. Furthermore, immune responses reciprocally recast metabolic regulation

Metabolic dysregulation is a defining characteristic of cancer cells and significantly influences the development and progression of CRC. Immunometabolism, the interplay between immune cell function and metabolism, is a critical determinant in cancer progression, particularly in the context of CRC [56]. Abnormal metabolic pathways of cancer include fatty acid, glucose, and amino acid metabolism. Other metabolic pathways include the one-carbon metabolism, pentose phosphate pathway, and nicotinamide adenine dinucleotide phosphate metabolism [57–60]. Metabolism and immunity are both important components in maintaining the normal operation of human body. They reinforce each other, and the components complement one another, as shown in Fig. 1. Glycolytic metabolism is the process of converting glucose uptake from the extracellular environment to pyruvate and releasing adenosine triphosphate (ATP) [61]. T-cell activation significantly increases glycolytic flux and transports glycolytic pyruvate into the tricarboxylic acid (TCA) cycle [62]. The metabolic profile of CD4^+^ T cells significantly influence their immune functions, which in turn, can modulate the pathogenesis of IBD [63, 64]. The macrophages undergo differentiation into either M1 or M2 cells [65].In M1 macrophages, the TCA cycle results in metabolite accumulation and enhances cell immune function. Fatty acid oxidation regulates the balance between inflammatory effector and suppressor T cells. Increased fatty acid oxidation and oxidative phosphorylation support Treg differentiation and function. Treg accumulates in inflamed tissues of colitis and is involved in the progression of CRC [66]. The differentiation of M2 macrophages also depends on the fatty acid oxidation program. The fatty acid synthesis pathway produces lipids, which are essential for cell growth and proliferation. Fatty acid synthesis also links innate and adaptive immunity by regulating DCs function. Amino acid metabolism is closely related to the mTOR pathway and nucleotide synthesis, and the metabolism of glutamine, arginine, and tryptophan regulates the activity of immune cells. The intricate metabolic demands shared by cancer and immune cells imply that effective targeting on cancer metabolism necessitates consideration of gene type, tumor type, and the composition of the tumor microenvironment. A comprehensive understanding of their respective roles and mechanisms is essential to realize the cancer metabolic therapy. The main regulation of immunometabolism in the TME involves the various critical signaling pathways in immunity. The phosphatidylinositol 3-kinase (PI3K)/AKT (also known as protein kinase B, PKB)/mammalian target of rapamycin (mTOR) and liver kinase B1-5’ (LKB1)-AMP-activated protein kinase (AMPK) signaling pathways are important in regulating immune metabolism [67]. The PI3K/AKT/mTOR signaling cascade is a critical cellular signaling pathway that governs a myriad of cellular processes, including cell growth, proliferation, metabolism, and survival. mTORC1 is highly activated in the intestinal mucosa of IBD patients, and inhibition of mTORC1 is effective in the treatment of UC [68]. mTORC1 subsequently activates the transcription factor hypoxia-inducible factor 1 (HIF1). In macrophages of IBD patients, glycolysis is significantly enhanced by mTORC1 and HIF-1 [69]. HIF-1 promotes glycolysis and cancer-related inflammation by stimulating hexokinase and pyruvate dehydrogenase kinase, co-inducing glycolytic gene expression with other oncogenes or transcription factors. On the flip side, glycolysis affects immature DCs (iDCs) [38, 67, 70, 71]. mTOR is an effector target of AKT signaling that increases glycolysis and reduces lipid oxidation. This pathway is essential for the differentiation of CD4^+^ T cells into immunologically specific effector T cells (Teff) or the induction of regulatory T-cell (Treg) subsets [72]. As an energy sensor in cells, AMPK activation reduces the levels of mitochondrial aerobic glycolysis and oxidative phosphorylation, and inhibits the migration, invasion, and metastasis formation of CRC cells [38, 73–76]. Targeting immunometabolism in the TME represents a highly promising therapeutic strategy [55, 77].

## Astragali Radix, Angelicae Sinensis Radix, and DGBX decoction advances

**Fig. 2:**
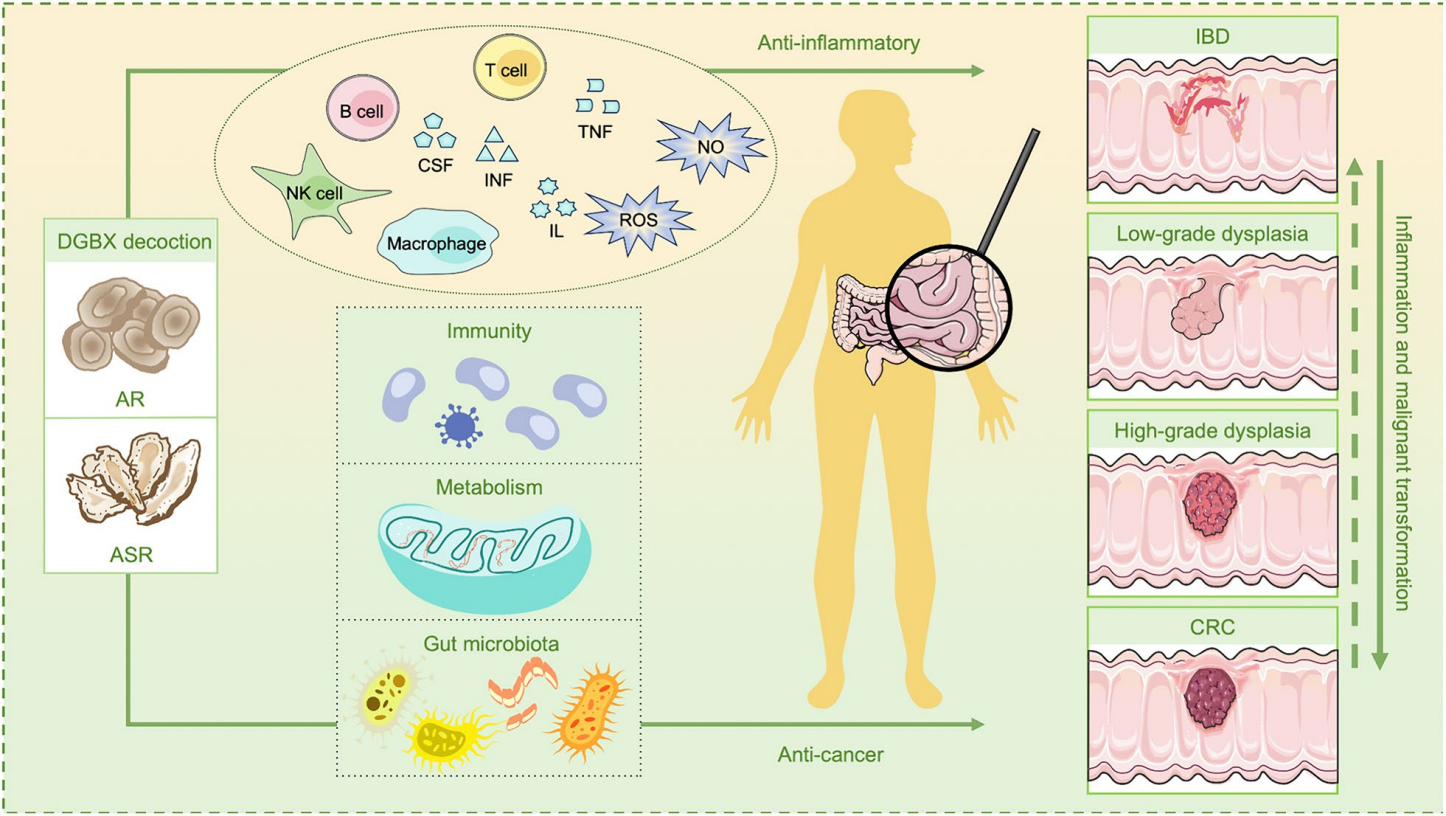
The scheme. DGBX decoction is composed of AR and ASR with a ratio of 5:1. AR and ASR both have anti-inflammatory and anti-cancer effects. Inflammation plays a pivotal role in the pathogenesis and progression of IBD, while anti-cancer effects show significant potential for CRC treatment. Hence, this review aims to comprehensively explore the therapeutic implications of DGBX decoction in IBD-associated CRC. *DGBX* Danggui Buxue, *AR* Astragali Radix, *ASR* Angelicae Sinensis Radix, *IBD* inflammatory bowel diseases, *CRC* colorectal cancer

Astragali Radix (AR, Huang Qi in Chinese), the dried root of *Astragalus membranaceus* (Fisch.), Bge. var. *mongholicus* (Bge.) Hsiao or *Astragalus membranaceus* (Fisch.) Bge., and the components isolated and identified included polysaccharides, saponins, flavonoids, and amino acids [78, 79]. As a traditional Chinese medicine employed in clinical treatment, AR exhibits diverse biological activities, including anti-inflammatory and anti-tumor functions [80–83]. Angelicae Sinensis Radix (ASR, Dang Gui in Chinese) is the root of *Angelica sinensis* (Oliv.) Diels [84]. The main chemical components of ASR include organic acids, volatile oil, polysaccharides, and flavonoids. It also has a variety of pharmacological activities, including anti-inflammatory activity, cardiac protection, antioxidant activity, and neuroprotection, as well as functioning in the cardiovascular and cerebrovascular systems [85, 86]. As a Chinese classical prescription, DGBX decoction is recorded with AR and ASR, in a ratio of 5:1. It is a classic recipe to invigorate Qi and tonify the blood [87, 88]. The main effective components in DGBX decoction are polysaccharides, calycosin, formononetin, astragaloside IV, ferulic acid, and ligustilide [89]. DGBX decoction exerts supporting Qi and enriches the blood, enhancing efficacy and reducing toxicity [90]. In recent years, traditional Chinese medicine and the classical prescriptions have been found to be widely used [83, 91], such as AR, ASR, and DGBX decoctions, especially for their anti-cancer activities, immune regulation, and metabolic regulation, as shown in Tables 1, 2 and 3. Accordingly, the schema of the present study is shown in Fig. 2.

**Table 1:** The main active ingredients derived from AR and ASR or the corresponding TCM prescriptions

Source	Active ingredients	Effects on CD/UC/CRC	Refs
*Astragalus membranaceus*	Calycosin	Attenuate TGF-β1 stimulation and ameliorates CD-induced intestinal fibrosis;	[92]
		Inhibit the growth and induces apoptosis of CRC cells;	[93]
	Formononetin	Formononetin significantly inhibits the growth and metastasis of CRC cells;	[94]
	Astragaloside II	Improve intestinal epithelial barrier function to alleviate CD;	[95]
		Alleviate DSS-induced UC in mice by reducing the level of inflammatory factors;	[96]
	Astragaloside IV	Regulate immune function and antioxidant stress, and alleviates DSS-induced UC in mice;	[97]
		Inhibit the growth and proliferation of CRC cells by regulating cell cycle;	[98, 99]
	Astragalus polysaccharide	Alleviate colonic mucosal injury and improve DSS-induced UC by regulating immune balance;	[100, 101]
		Attenuate inflammation by regulating cytokines and improves DSS-induced UC;	[102]
		Protect intestinal mucosa by regulating cytokines and improves DSS-induced UC;	[103]
Aidi injection (including *Mylabris phalerata* Pallas, *Astragalus membranaceus* (Fisch.) Bge., *Panax ginseng* C. A. Mey., and *Acanthopanax gracilistylus* W.W. Smith)	Calycosin-7-O-β-D-glucoside	Induce apoptosis synergistically inhibits the growth of colorectal cancer;	[104]
Danggui Buxue	Danggui Buxue Decoction	Inhibit the growth of CRC cells and induced autophagy;	[105]
		Induce apoptosis of tumor cells and alleviates metastatic CRC;	[106]
Angelicae Sinensis	ASR extract	Deplete ROS to resist oxidative stress and treat AOM/DSS-induced CRC	[107]
	z-ligustilide	Decrease the viability of CRC cells and inhibits their proliferation;	[108]
	Angelica sinensis polysaccharide	Inhibit myeloperoxidase activity and reduces proinflammatory cytokine levels to combat DSS-induced UC;	[109]
		Protect against oxidative stress and DNBS-induced acute UC;	[110]

**Table 2:** Immunomodulatory effects of active ingredients derived from AR and ASR or the corresponding TCM prescriptions

Source	Active ingredients	Action and Mechanism	Refs
Astragali Radix	Calycosin	Reduce migration of macrophages to endothelial cells, which plays a key role in diabetes;	[145]
	Formononetin	Reduce acute lung injury caused by hyperoxia in mice by reducing M1 macrophages and increasing M2 macrophages;	[146]
	Astragaloside IV	Inhibit M2 macrophage polarization by inhibiting AMPK signaling, thereby inhibiting tumor growth and metastasis;	[116]
		Attenuate the severity of autoimmune encephalomyelitis disease by inhibiting DC maturation and function;	[147]
	Astragaloside IV	Increase T lymphocyte proliferation, inhibit IL-1 production and decreased TNF-α activity;	[148]
	Astragalus polysaccharide	Promote the differentiation and maturation of DC and enhance adaptive antitumor immune response;	[149, 150]
		Regulate the differentiation of Tfh subsets in colitis mice, inhibit the response of Tfh cells, improve the function of Treg cells, and ameliorates UC by regulating the balance between Tfh and Treg cells;	[101]
*Astragalus membranaceus*	Astragaloside IV	Inhibit Tfh cell differentiation in vivo, expand TFR cell response, and improve pulmonary hypertension;	[151]
		Induce the polarization of M2 macrophages into M1 macrophages, resulting in a significant reduction of M2 and an increase of M1 phenotype;	[152]
		Restore immune homeostasis and alleviate DSS-induced colitis by reshaping the balance of Th17 / Treg cells;	[97]
	Astragalus polysaccharide	Activate lymphocytes and improves immunity, upregulate the expression of IL-2, TNF-α and IFN-γ in peripheral blood, and enhance anti-tumor defense;	[153]
		Enhance M1 polarization, increase the ratio of M1/M2 macrophages in cells, and inhibited tumor growth;	[115]
Astragalus mongholicus	Astragalus polysaccharide	Induce the production of NO in macrophages to kill tumor cells;	[154]
modified Jian-pi-yang-zheng (mJPYZ, including Astragalus mongholicus (Huangqi))	Calycosin, Formononetin	Decrease the level of aerobic glycolysis in gastric cancer cells through PKM2/HIF-1α, regulate tumor-associated macrophages, and inhibited the proliferation, migration and invasion of gastric cancer cells;	[155]
*Ligusticum chuanxiong*	Astragaloside IV	Attenuate DSS-induced colitis by differentiating immature macrophages into mature macrophages through STAT1 signaling;	[156]
Bu-Shen-Yi-Qi formulae (BSYQF, including *Astragalus membranaceus* (Fisch.) Bunge)	Astragaloside II, Astragaloside IV, Calycosin, Formononetin	Reduce neutrophils and lymphocytes, reduce airway inflammation and treat asthma by regulating the balance of Treg/Th17 cells;	[157, 158]
Bushen Huoxue recipe (BHR, including 16.7% *Astragalus menbranaceus* Bunge. (Milkvetch Root), 8.3% *Angelica sinensis* Diels (Angelicae Sinensis Radix))	Calycosin, Ferulic acid	Attenuate ovarian dysfunction by reducing CD4 T cells, Th1 and Th17 cells;	[159]
*Astragalus membranaceus* and *Panax ginseng*	Formononetin	Regulate immunity and improves adaptive immunity;	[160]
Buyang Huanwu Decoction (BYHWD, including Astragali Radix (120 g), Angelicae Sinensis Radix (6 g))	Ferulic acid, Calycosin, Formononetin	Reduce the proliferation of rod cells induced by Con A, and had anti-inflammatory and vasodilator effects;	[161]
Dahuang Zhechong pill (DHZCP)	Formononetin	Regulate the immune status of liver cancer mice by decreasing Treg and increasing Th1 cell level;	[162]
Angelicae Sinensis	Ligustilide	Reduce IL-6 production in cells, inhibit macrophage recruitment and M2 polarization, and attenuate cancer progression;	[163]
	Angelica sinensis polysaccharide	Induce splenocyte proliferation in vitro and stimulate peritoneal macrophages to secrete soluble factors, which play an important role in tumor development;	[164]
		Activate a variety of immune cells, include promoting the proliferation of MDSC and enhance its immunosuppressive function in a concentration-dependent manner;	[165]
		Increase the number of peritoneal macrophages and T cells, and promote the secretion of cytokines IL-2 and IFN-γ;	[166]
		Promote the phagocytosis of peritoneal macrophages and the killing activity of NK cells, and induce a protective immune response against leukemia;	[127, 129, 167]
*Radix Angelicae sinensis* and *Ligusticum chuanxiong*	Ligustilide	Increase the expression level of anti-inflammatory cytokines in Treg cells and suppresse neuroinflammation by regulating adaptive immunity;	[168]
*Angelica acutiloba*	Z-ligustilide	Inhibit IL-6 and TNF-α to exert anti-inflammatory activity;	[169]

**Table 3:** Metabolic regulation of active ingredients derived from AR and ASR or the corresponding TCM prescriptions

Source	Active ingredients	Effects	Mechanisms	Refs
Astragali Radix	Calycosin	Inhibit oxidative stress and improve autophagy	AMPK/SKP2/CARM1 signaling pathway	[192]
	Calycosin-7-O-β-D-glucoside	Attenuate lipid accumulation	AMPK signaling pathway	[193]
	Calycosin	Regulate lipid metabolism and increase fatty acid β-oxidation	Farnesoid X receptor	[194]
		Regulate lipid metabolism, inhibit fat generation, and promote fat decomposition	mTOR/autophagy pathway	[170]
	Formononetin	Protect mitochondrial membrane integrity	ROS signal, PI3K/Akt signaling pathway	[195]
		Anti-inflammatory and reduces muscle atrophy	PI3K/Akt/FoxO3a pathway	[160]
	Astragaloside IV	Promote fatty acid oxidation and improve lipid metabolism	AMPK/ACC1/mitochondrial β-oxidation signal axis	[196]
		Inhibit lipid production and reduce lipid accumulation	AMPK pathway	[197]
		Reduce triglyceride ester, alleviate lipid metabolism disorder	PI3K/AKT pathway	[175]
		Reduce lipid accumulation	ROS signal	[198]
	Astragaloside A	Enhance fatty acid oxidation and regulate energy metabolism	Peroxisome proliferator-activated receptor alpha (PPARα)	[199]
	Astragalus polysaccharide	Increase glucose uptake	AMP-AMPK-AS160 pathway	[200]
		Enhance autophagy level	PI3K/AKT/mTOR pathway	[178]
		Inhibition of oxidative stress	PI3k/Akt pathway, p38MAPK pathway	[201]
		Regulate glucose and lipid metabolism	STRs pathway	[202]
		Regulate lipid accumulation	SCFA-GPR signaling pathway	[203]
		Enhance glucose uptake	AMPK pathway	[204]
Angelicae Sinensis Radix	Ligustilide	Inhibition of glycolytic metabolism	PTEN/AKT signaling	[205]
	Angelica sinensis polysaccharide	Relieve lipid disorder and improve oxidative stress	Adiponectin-SIRT1-AMPK signaling	[206]
		Regulate glucose metabolism	PI3K/AKT pathway	[207]
		Regulate the metabolism of glycine and arachidonic acid	——	[208]
		Regulate lipid metabolism and amino acid metabolism	——	[209]
	Z-ligustilide	Regulate oxidative stress	PI3K/Akt pathway	[210]

### AR and ASR exhibit promising anti-inflammatory properties

By subcutaneous injection of air and Zymosan solution into the back of mice, a Zymosan air–pouch mouse model was established to induce inflammation. The higher dose of aqueous AR extract (100 mg/kg) effectively inhibited the expression of interleukin (IL)-1β, IL-6, and tumor necrosis factor (TNF)-α, indicating its anti-inflammatory effect through suppression of pro-inflammatory cytokines production. In addition, in lipopolysaccharide (LPS)-induced inflammation of RAW 264.7 cells, AR was found to inhibit the synthesis of inflammatory mediator nitric oxide (NO) and the expression of nitrite oxide synthase (iNOS) [111]. Astragalus polysaccharides and astragaloside IV are the primary bioactive compounds extracted from AR. Astragaloside IV enhances the tyrosine phosphatase activity of CD45 protein to induce T-cell activation, manages the balance of Teff/Treg cells to regulate immunity, and inhibits pro-inflammatory cytokines and nuclear factor-κB (NF-κB) pathways to enhance anti-inflammatory activity [112, 113]. In an orthotopic implantation lung cancer model utilizing C57 BL/6 mice, which was established using 3LL-LUS-IDO cells, astragaloside IV, administered at a dosage of 40 mg/kg, has been demonstrated to effectively suppress the expression of indoleamine 2,3-dioxygenase in vivo. It also down-regulates the population of Tregs while concurrently up-regulating the activity of cytotoxic T lymphocytes to enhance the immune response, thereby showcasing anti-cancer activity [114]. By culturing human lung cancer cells and human mononuclear cells in vitro, it was found that astragaloside IV at a dosage of 40 mg/kg, significantly inhibits the M2 macrophage polarization of tumor-associated macrophages (TAMs) through the modulation of AMPK signaling pathway. This finding was corroborated through parallel experiments conducted on primary human macrophages, which further substantiate the immunomodulatory role of astragaloside IV in regulating macrophage function within the tumor microenvironment [115, 116]. Astragalus polysaccharides, administered at a dosage of 3 mg/kg, exert comparable effects on a lung cancer subcutaneous model in vivo, enhancing the anti-cancer efficacy of cisplatin by modulating the activity of inflammation-associated macrophages. The anti-inflammatory effects of astragalus polysaccharides and astragaloside IV on bovine mammary epithelial cells induced by LPS were also studied. Bovine mammary epithelial cells stimulated with LPS were utilized as an in vitro model of inflammation to investigate the impact of astragalus polysaccharides (an efficacious concentration is 100 μg/mL) and astragaloside IV (an efficacious concentration is 1 mg/mL) on inflamed bovine mammary epithelial cells. It was found that both could significantly reduce the relative expression of IL-6, IL-8, and TNF-α, and activate the Wnt/β-catenin signaling pathway to inhibit inflammation [117]. Atragaloside IV also exerts inhibitory effects on the TLR4/NF-κB signaling pathway and the activation of autophagy, thereby attenuating cellular inflammation by reducing the release of inflammatory mediators [118]. CT26 cells were orthotopically implanted into BALB/c mice to establish a subcutaneous tumor model. Astragaloside III, administered at a dosage of 50 mg/kg in five bi-daily treatments, significantly activated NK cells in tumor environment, thereby enhancing the cytotoxic capacity of NK cells and leading to a notable inhibition of tumor growth. Further assay via co-culture of NK cells with CT26 cells revealed that astragaloside III up-regulated the expression of NK group 2D, Fas and IFN-γ in NK cells, thereby exerting a pronounced suppressive effect on the proliferation of CT26 colorectal tumor cells [119]. Flavonoids isolated from AR alleviate DSS-induced colitis by enhancing mitophagy levels, inhibiting NLRP3 inflammasome activation, and reducing the production of pro-inflammatory cytokines in colon tissue [120].

Calycosin is the predominant isoflavonoid in AR. Calycosin, administered at a dosage of 4.67 mg/kg, effectively reduces the levels of TNF-α and IL-1 in the serum of rats with heart failure induced by ligation of the left anterior descending artery, indicating that calycosin could alleviate the inflammatory response in rats with heart failure. In vitro cardiomyocyte cultures showed that calycosin exerts anti-inflammatory effects via the PI3K-AKT signaling pathway [121]. In glucocorticoid-induced osteonecrosis of the femoral head in rats, calycosin, administered at a dosage of 10 mg/kg, promotes bone formation, inhibits the TLR4/NF-κB pathway, and significantly regulates inflammation, thus effectively alleviating osteonecrosis of the femoral head. In addition, calycosin also inhibits LPS-activated inflammation in vitro by inhibiting the TLR4/NF-κB pathway [122]. Formononetin, a naturally occurring flavonoid derived from AR, has been reported to have immunomodulatory effects [123]. By pre-treatment of LPS-induced mastitis model mice with formononetin, administered at dosages of 10, 20 and 30 mg/kg, myeloperoxidase activity was reduced along with TNF-α and IL-1β production. In vitro experiments using EpH4-Ev cells from mouse mammary epithelial cells stimulated with LPS showed that formononetin, administered at dosages of 10, 20 and 30 μM, inhibits LPS-induced activation of the NF-κB signaling pathway [124]. Taken together, the active component from AR effectively modulates immune cells and cytokines to alleviate inflammatory symptoms.

ASR is also an herb used to regulate the immune system, and its active ingredient acts as an antioxidant and anti-inflammatory agent. Angelica sinensis polysaccharide, extracted from the roots of ASR, is a β-D-pyranoid polysaccharide. It is also a crucial herbal constituent in various traditional formulations utilized for the management of inflammatory responses [125]. Four polysaccharides extracted from different roots of Angelica sinensis have anti-inflammatory activity on intestinal epithelial system, and their activity varies with the difference of structure [126]. Angelica sinensis polysaccharide, administered at a dosage of 40 mg/kg, significantly reduced the levels of TNF, IF-2 and interferon-γ(IFN-γ) in L1210-bearing mice. In addition, angelica sinensis polysaccharide increased the number of lymphocytes, enhanced the ability of macrophages and natural killer cells, and induced a protective immune response [127]. Angelica sinensis polysaccharide, administered at a dosage of 6 mg/kg, significantly reduces the levels of TNF-α, IFN-γ, IL-2, and IL-6 in concanavalin A-induced mouse hepatitis models [128]. Both astragalus polysaccharides and angelica sinensis polysaccharide increase the levels of IL-2 and TNF-α in H22 tumor-bearing mice. Astragalus polysaccharides, administered at a dosage of 400 mg/kg, enhance the phagocytic function of peritoneal macrophages in H22 tumor-bearing mice, while angelica sinensis polysaccharide, administered at a dosage of 200 mg/kg, enhance the activity of T, B lymphocytes, and NK cells, and improve the proportion of lymphocyte subsets in the peripheral blood of H22 tumor-bearing mice. Both significantly inhibit tumor growth in mice [129, 130]. Ligustilide is a bioactive phthalide derivative isolated from ASR, which significantly improves the infiltration of peripheral immune cells, inhibits Th1 immunity, increases Th2 immunity, and re-establishes Th1/Th2 balance [131, 132]. Treatment of human umbilical vein endothelial cells with ligustilide, administered at dosages of 1, 3, 10 μM, significantly inhibits TNF-α and activates the Nrf2/HO-1 signaling pathway, alleviating vascular inflammation, and protecting the blood vessels [133]. Ferulic acid is a phenolic acid isolated from ASR, which has a variety of biological activities, including regulation of inflammation. Ferulic acid was found to improve hepatic oxidative stress and inflammation by activating AMPK in mouse hepatic fibrosis induced by carbon tetrachloride and LPS-induced macrophage inflammation [134]. At an efficacious concentration of 20 μM, ferulic acid inhibits LPS-induced expression of pro-inflammatory cytokines, including TNF-α, IL-6, and IL-1β, and ROS production in macrophages by blocking NLRP3 inflammasome activation [135]. Furthermore, within the concentration range of 1, 2, 4 mM, ferulic acid dose-dependently down-regulates the expression of LC3-II, Beclin 1 and Atg12-Atg5 complex. This modulation of autophagy contributes to its efficacy as an anti-cancer agent by inhibiting the autophagic flux [136]. Additionally, tributyltin ferulate, a derivate of ferulic acid with an efficacious concentration of 400 nM, has been demonstrated to induce autophagic cell death in HCT-116 colon cancer cells, thereby exhibiting anti-tumor properties [137]. Therefore, ASR also effectively mitigates inflammation and modulates immune responses.

**Fig. 3:**
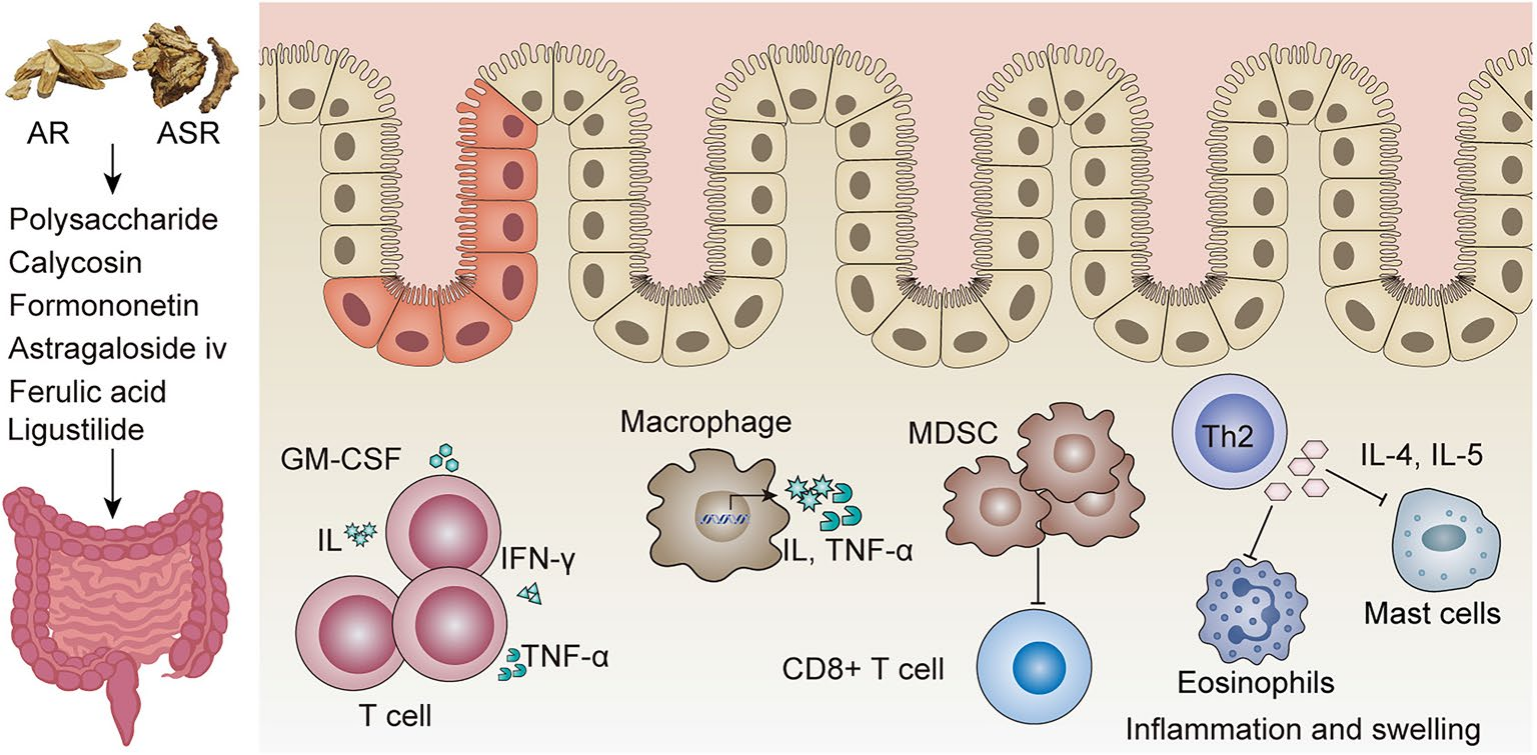
The anti-inflammatory activities of DGBX decoction. DGBX decoction contains polysaccharide, calycosin, formononetin, astragaloside IV, ferulic acid, and ligustilide. These active ingredients interfere with immune cells and modulate cytokines through various signaling pathways to attenuate inflammation

Regarding the aspect of inflammation modulation, DGBX decoction regulates immune responses and improves inflammatory symptoms, as shown in Fig. 3. For T lymphocytes, DGBX decoction induces cytokines released from T cells, such as interleukin (IL), granulocyte–macrophage colony-stimulating factor (GM-CSF), IFN-γ, and TNF-α. Phosphorylation of extracellular signal-regulated kinase (ERK) 1/2 is induced to stimulate T lymphocyte proliferation. For macrophages, DGBX decoction treatment increases phagocytosis [138, 139]. Polysaccharides in DGBX decoction induce IκBα degradation, and activate NF-κB signaling pathways, stimulating the immune response. In macrophages, DGBX decoction exerts a pivotal role in host defense mechanisms by dose-dependent suppression of the expression of pro-inflammatory cytokines IL-1β, IL-6, and tumor necrosis factor at both mRNA and protein levels [140]. DGBX decoction significantly reduces the production of pro-inflammatory cytokines, and effectively improves the inflammatory state and pathological structure of DSS-induced IBD model, promoting inflammation resolution. MDSC inhibits the functional activity of CD8^+^ T activity and improves intestinal inflammation, and DGBX significantly increases the level of MDSC to change the composition of intestinal mucosal immune cells eventually. At the same time, it boosts the proliferation of intestinal epithelial cells and facilitates swift repair of damage to the intestinal mucosal barrier [141, 142]. DGBX decoction attenuates tubulointerstitial fibrosis in rats with unilateral ureteral obstruction by inhibiting the expression of NOD-like receptor family Pyrin domain 3 (NLRP3) inflammasome and significantly reduces the expression of α-smooth muscle actin (α-SMA) representative protein [143]. In 2,4-dinitrochlorobenzene induced mice atopic dermatitis, DGBX decoction significantly inhibits excessive production of IL-4 and IL-5 by Th2 cells, along with a notable reduction in eosinophil and mast cell infiltration, thereby mitigating inflammation and swelling [144]. The potential impact of DGBX decoction on inflammation and immunity is supported by its anti-inflammatory and immunomodulatory effects, mediated by the AR and ASR constituents. Further experimental validation is required to substantiate the immunometabolism potential.

### Metabolism modulation aspect

Abnormal metabolism of cancer has highlighted therapeutic targets in recent years. Glucose and amino acids uptake, nutrition acquisition preference, the metabolic intermediates, even the metabolite-driven gene regulation, have been highlighted to explore the novel treatments or targets [57]. AR and ASR both interfere with cell metabolism and improve blood lipids and blood glucose by regulating abnormal cellular metabolic pathways, including fatty acid metabolism and glucose metabolism. AR extract significantly reduces HFD-induced lipid storage, increases the processes of lipolysis and lipid β-oxidation, and alleviates acquired hyperlipidemia in HFD-fed mice by regulating lipid metabolism [170]. Based on pharmacology network analysis and experimental verification, it was found that AR water extract stimulates fat cells and promotes fatty acid metabolism to maintain fatty acid homeostasis [171]. Astragalus polysaccharides at a dosage of 0.25 g/kg regulate cholesterol homeostasis by reducing plasma total cholesterol (TC), triglycerides (TG), and low-density lipoprotein cholesterol (LDL-C) in hypercholesterolemia hamsters [172]. Meanwhile, astragalus polysaccharides (700 mg/kg) regulates blood glucose in insulin resistant C57BL/6 J mice by alleviating ER stress [173]. Astragaloside IV, administered at a dosage of 80 mg/kg, alleviates hepatic injury in type 2 diabetes mellitus rats by modulating the AMPK/mTOR pathway, also attenuating dyslipidemia, oxidative stress, and inflammation [174]. Additionally, astragaloside IV, administered at a dosage of 50 mg/kg, exerts hypoglycemic effects in a rat model of diabetes induced by a high-sugar diet combined with streptozotocin by modulating intestinal microbiota [175]. Calycosin-7-glucoside, administered at a dosage of 0.05 mg per mouse, inhibits glycolysis in the db/db mouse model of diabetes mellitus through the activation of AMPK pathway in an inflammatory environment, reducing the inflammatory response and promoting healing of diabetic wounds [176]. Abnormal metabolism in cancer results in different phenotypic characteristics from normal cells, including cell proliferation, migration, invasion, and angiogenesis [177]. Calycosin and Astragaloside IV both inhibit transforming growth factor-β (TGF-β). Calycosin inhibits colorectal cancer cell growth through the PI3K/AKT pathway, upregulates basic leucine zip-ATF-like transcription factor 2 (BATF2) and downregulates plasminogen activator inhibitor-1(PAI-1), and inhibits TGF-β-induced cell migration and enhances the effect of TGF-β induction on cell apoptosis. The mechanism of regulating autophagy is related to the PI3K/AKT/mTOR signaling pathways. Astragalus polysaccharides reduce the levels of p-AKT and p-mTOR in cells, block PI3K/AKT/mTOR signaling pathways, increase autophagy, and alleviate inflammation, to effectively suppress gastric cancer [178–181]. Angelica sinensis polysaccharide ameliorates the inflammatory response in PC12 cells induced by LPS, attenuates cellular apoptosis, and mitigates cellular damage by down-regulating COX-1 expression and the activation of PI3K/AKT signaling pathway [182]. In addition, Astragaloside IV regulates AMPK, NF-κB, and signal transducer and activator of transcription (STAT) signaling pathways, inhibits the polarization of M2 macrophages, and reduces the progression and metastasis of liver cancer cells and lung cancer cells [116, 183, 184]. Both Astragaloside IV and ligustilide alleviates experimentally DSS-induced colitis. Astragaloside IV, administered at dosages of 50 and 100 mg/kg, effectively inhibits the polarization of M1 macrophages and ameliorates colitis through modulation of STAT signaling pathway. Astragalus saponins reduces the expression level of glycolytic enzymes to attenuate aerobic glycolysis and inflammation, inhibiting colitis eventually. Ligustilide, administered at dosages of 15, 30 and 60 mg/kg, activates peroxisome proliferator-activated receptor γ (PPARγ) and inhibits NF-κB and AP-1 signaling, controlling the expression of pro-inflammatory cytokines IL-1β, IL-6, and TNF-α to alleviate experimental colitis in mice. [156, 185, 186]. ROS are byproducts of cellular metabolism, and the ROS level of cancer cells is higher than that of non-tumor cells. Formononetin mitigates cisplatin-induced nephrotoxicity in LLC-PK1 porcine kidney epithelial cells by suppressing intracellular ROS accumulation and oxidative stress [187]. Similarly, angelica sinensis polysaccharide also inhibits oxidative stress in vivo and in vitro, decrease superoxide dismutase (SOD) activity, and improve acetaminophen-induced acute liver injury to achieve liver protection. Ferulic acid has antioxidant activity, while tributyltin ferulate stimulates ROS production, leading to autophagy activation, showing an obvious anti-tumor effect in colon cancer cells [137, 187, 188]. Astragalus polysaccharides, administered at a dosage of 200 mg/kg, regulate the intestinal microenvironment, including regulating the composition of the intestinal microbiota and its metabolic function, changing the composition of fecal metabolites, reducing the expression levels of IL-1β and IL-6 in serum, weakening the immunosuppressive activity of MDSC, and inhibiting the growth of melanoma in mice [189]. DGBX decoction induces ROS production in the mitochondria of osteoblasts, thereby activating the AMPK pathway, affecting glycolytic capacity, and improving bioenergy [190]. In addition, the potent cardioprotective effect of DGBX decoction is mediated by the regulation of mitochondrial bioenergetics to improve the health status of H9C2 cardiomyoblasts [191]. In conclusion, DGBX decoction and its principal constituents actively participate in metabolic regulation, modulate immune pathways, exerting a therapeutic effect.

### Anti-cancer aspect

AR is a traditional tonic herb widely used in the treatment of various cancers. AR aqueous extracts were applied to different cancer cell lines and were found to inhibit a variety of cancer cell growths [211]. AR and its four major bioactive compounds, including calycosin, formononetin, astragaloside IV, and astragalus polysaccharides, were found to have effects on breast cancer cells. Calycosin, at efficacious concentrations of 200 and 400 μM, impedes the migration and invasion of breast cancer cells by suppressing the epithelial-mesenchymal transition process. Formononetin reduces autophagy by regulating mTOR, promotes apoptosis of paclitaxel-resistant triple-negative breast cancer cells, and overcomes paclitaxel resistance [212]. The combination treatment involving formononetin at efficacious concentrations of 40 and 80 μM, in conjunction with metformin, exerts synergistic inhibition of MCF-7 breast cancer cells proliferation and induces apoptosis. Through MDA-MB-231 breast cancer cells in vitro experiments and orthotopic mouse tumor models for in vivo experiments, astragaloside IV was found to inhibit cell viability and invasion of breast cancer cells. Astragalus polysaccharides, administered at concentrations of 100, 200, 500 and 1000 μM, did activate the macrophage-like RAW 264.7 cells in in vitro models to induce apoptosis, thereby inhibiting the viability of MCF-7 cells [78, 213–216]. Calycosin and astragaloside IV shows anti-tumor activity against CRC and gastric cancer cells. Calycosin, administered at concentrations of 25, 50 and 100 μM, significantly induces apoptosis in HCT116 cells and inhibits cell proliferation and invasion in a dose-dependent manner. Calycosin exhibits significant cytotoxicity against AGS cells, with an IC50 value of 47.18 ± 1.27 μM, while demonstrating minimal toxicity towards normal cells. Astragaloside IV exhibits a dose-dependent inhibition of proliferation in both SW620 and HCT116 cells, while it had no significant effect on the proliferation of normal colonic fetal human cells. N-methyl-N'-nitro-N-nitrosoguanidine was used to induce gastric precancerous lesions (GPL) in a model. Astragaloside IV, at efficacious concentrations of 50 and 100 mg/kg, has been demonstrated to modulate autophagy and apoptosis, thereby exerting a protective effect on gastric mucosal injury and improving both intestinal metaplasia and dysplasia within precancerous gastric lesions [98, 217–219]. Astragalus polysaccharides have been shown to participate in a variety of biological processes, encompassing inflammation, metabolism, and carcinogenesis. Cell experiments have shown that astragalus polysaccharides reduce prostate cancer cell proliferation and lipid metabolism in a dose-dependent manner. Utilizing a tumor xenograft model, astragalus polysaccharides, administered at a dosage of 100 mg/kg, have been shown to exert an inhibitory effect on tumor growth via modulation of the miR-138-5p/SIRT1/SREBP1 signaling pathway [220]. Angelica sinensis polysaccharides obtained from ASR are primarily composed of arabinose, glucose, and galactose. Angelica sinensis polysaccharide, at efficacious concentrations of 25, 50, and 100 mg/kg, significantly inhibits tumor growth in H22 tumor-bearing mice by suppressing the production of hepcidin, thereby reducing intracellular iron concentration [221]. Ferulic acid shows inhibitory effects on both Hela and Caski cervical cancer cell lines. By downregulating the expression of MMP-9, ferulic acid suppresses cell invasion in cervical cancer cells. Moreover, ferulic acid inhibits autophagy by decreasing the levels of related proteins LC3-II, Beclin-1, and Atg12-Atg5 in a dose-dependent manner [136]. Ligustilide and two other phthalides extracted from ASR have cytotoxic and anti-proliferative effects on HT-29 [108]. Ligustilide can alter the immunosuppressive function of cancer-associated fibroblasts. Cellular experiments show that ligustilide significantly inhibits prostate cancer and prostate cancer-associated fibroblasts and induces apoptosis of prostate cancer-associated fibroblasts through the TLR4 pathway [222, 223].

**Fig. 4:**
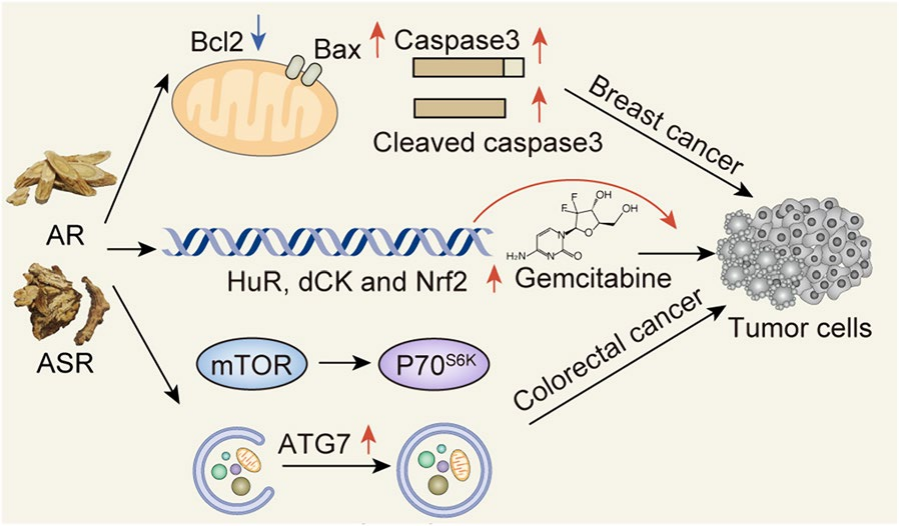
The anti-tumor activity of DGBX decoction. DGBX decoction regulates apoptotic proteins to induce apoptosis of breast cancer cells. Modulation of HuR, dCK and Nrf2 proteins alleviates the suppression of gemcitabine and enhances the anti-cancer effect of gemcitabine. Modulation of autophagic pathways has been shown to induce autophagic cell death in colorectal cancer cells

DGBX decoction influences tumor development, including inducing cell apoptosis and inhibiting metastasis, enhancing immune function, improving chemotherapy sensitivity, and reducing bone marrow suppression, as shown in Fig. 4. Myelosuppression is a frequently encountered adverse effect of most chemotherapy drugs. In gemcitabine-induced myelosuppression mice, DGBX decoction enhances the anti-cancer effect of gemcitabine by regulating the expression of stress response protein Hu antigen R (HuR), deoxycytidine kinase (dCK), and nuclear factor erythroid 2-related factor (Nrf2). Meanwhile, it inhibits the proliferation of cancer cells, increases the number of bone marrow nucleated cells and the level of hematopoietic cytokine thrombopoietin to alleviate myelosuppression induced by gemcitabine, and improves hematopoietic function [224]. In addition, the combination of DGBX decoction and gemcitabine enhances anti-cancer activity, represented by the increased level of granulocyte–macrophage colony-stimulating factor (GM-CSF), the enhanced immune ability, increased deoxycytidine kinase (dCK), and decreased P-glycoprote in a murine lewis lung carcinoma model [225]. Polysaccharide-depleted DGBX decoction partially inhibits the cell viability of colorectal adenocarcinoma cells, enhances the proliferation inhibition effect of 5-fluorouracil (5-FU), induces apoptosis, and increases sensitivity to chemotherapy or radiotherapy [105]. In addition, phase II clinical studies have shown that DGBX decoction prevents chemotherapy-induced myelosuppression in breast cancer patients [226]. According to network pharmacological analysis, 28 active compounds of DGBX decoction were predicted to hit 61 common targets. CT26 cells were employed to develop a murine model of metastatic colon cancer in BALB/c mice. In vivo experiments showed that DGBX decoction alleviates the progression of metastatic breast cancer by upregulating the expression of pro-apoptotic proteins Bax, inducing the activation of Caspase-3, and downregulating the expression of anti-apoptotic protein Bcl-2 to induce apoptosis [106]. DGBX decoction induces autophagic death of colorectal cancer cells and inhibits the growth of colorectal adenocarcinoma by regulating the mTOR/P70^S6K^ signaling pathway and upregulating autophagy related protein 7 (Atg7) [227]. DGBX decoction, particularly its polysaccharide-depleted fraction, potentiates the growth inhibitory effects of 5-fluorouracil and radiation treatment, possibly by inducing autophagy [105]. DGBX decoction also regulates intestinal flora, enhances immunity of mice by regulating *Lactobacillus* and *Odoribacter*, and reduces cancer-related bacteria such as *Helicobacter* and *Lactococcus*, showing anti-tumor activity [228].

## Immunometabolism potential of DGBX decoction in IBD-related CRC

### Promoting intestinal mucosal repair

**Fig. 5:**
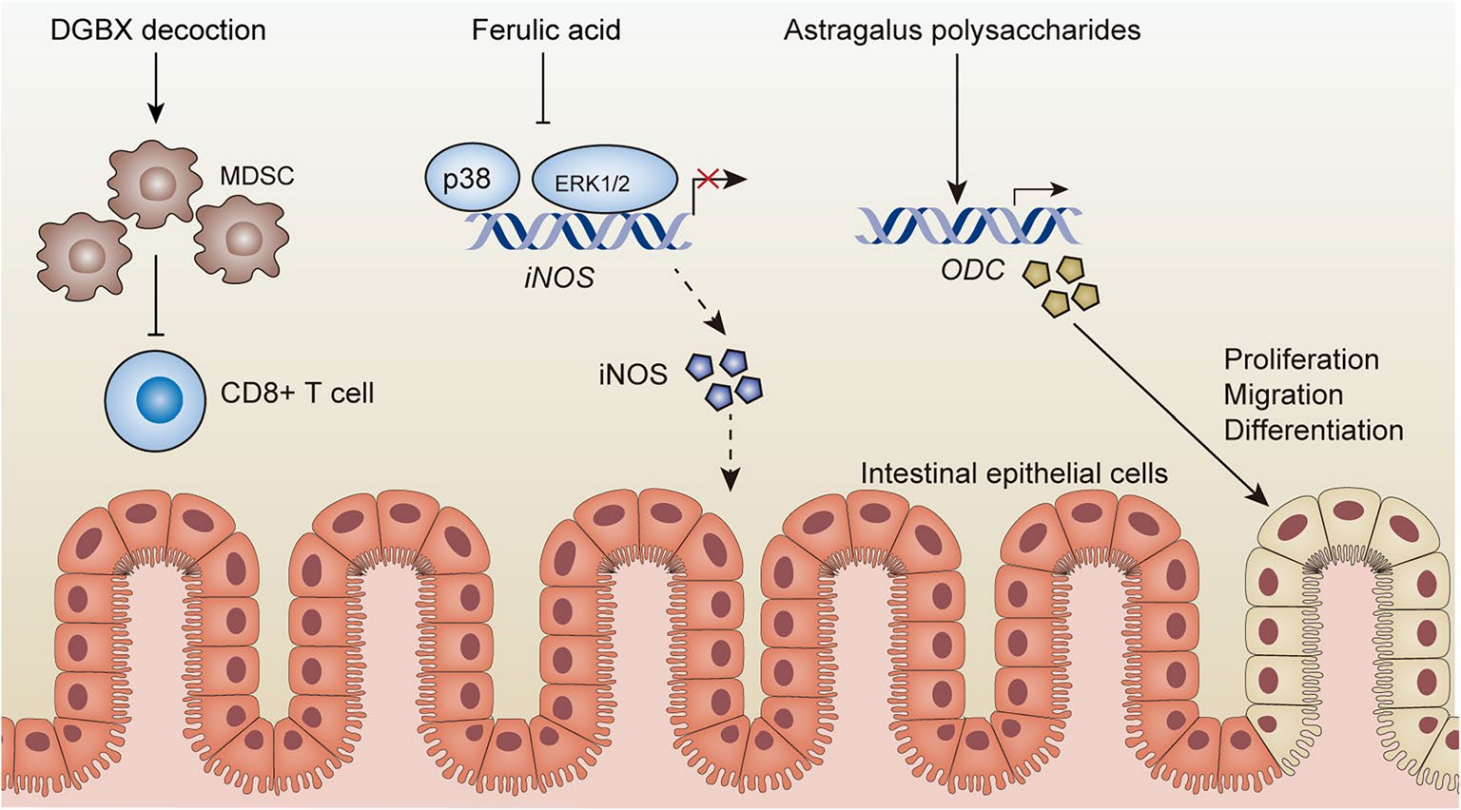
Effects of DGBX decoction and its principal constituents on intestinal barrier. DGBX decoction increases MDSC immune cells and inhibits the activity of CD8 +T cells. Ferulic acid inhibits the expression of MAPK p38, ERK1/2 and iNOS. Astragalus polysaccharides stimulates ODC gene synthesis of polyamine organisms, which promotes the proliferation of intestinal epithelial cells and improves inflammatory symptoms

The intestinal mucosal barrier is essential to prevent bacterial invasion and maintaining intestinal homeostasis. Intestinal epithelial cells and the tight junction complex between epithelial cells serve as mechanical barriers. The disruption of the intestinal mucosal barrier may result in bacteria and toxins invading normal colon tissue, causing local inflammation, and promoting its carcinogenic transformation [229]. AR has the effect of reducing intestinal inflammation. AR extract, administered at dosages of 5, 10, 50 and 100 μg/mL, reduces the expression of TNF-α and the activation of NF-κB, alleviates the inflammatory response of intestinal epithelial cells, and inhibits the destruction of the intestinal mucosal barrier and the increase of permeability caused by inflammation [230]. AR decoction reduces the levels of inflammatory factors, improves the intestinal mucosal injury induced by lipopolysaccharides in mice, and promotes tissue repair [94]. In addition, astragalus polysaccharides promote the proliferation of intestinal epithelial cells in vitro in a dose-dependent manner. Astragalus polysaccharides stimulates the ornithine decarboxylase (ODC) gene to synthesize polyamine organisms and promote the proliferation, migration, and differentiation of intestinal epithelial cells [231]. Astragaloside IV, administered at a dosage of 3 mg/kg, has been demonstrated to attenuate intestinal mucosal injury induced by sepsis through the downregulation of the RhoA/NLRP3 inflammasome signaling pathway [232]. When administered at the early stage of an AOM/DSS model, ASR extract was found to reduce DNA damage and exert an antioxidant effect in epithelial tissues [107]. In rats with 2,4-dinitrobenzene sulphonic acid (DNBS)-induced acute UC, the content of glutathione was decreased by angelica sinensis polysaccharide, and the protective effect on the intestinal mucosa may be attributed to oxidative stress [110]. Ferulic acid, administered at a dosage of 1 μM, can reduce the LPS-induced inflammatory response in human intestinal epithelial model Caco-2 cells, inhibit the activation of MAPK p38 and ERK1/2, inhibit the expression of iNOS, and alleviate intestinal inflammation [233]. DGBX decoction was found to repair intestinal mucosal barriers and improve IBD. DGBX decoction inhibits the activity of CD8^+^ T cells by increasing the number of MDSC immune cells, to improve intestinal inflammation. DGBX decoction treatment not only regulates immunity, but also promotes the repair of intestinal mucosal damage by accelerating the proliferation of intestinal epithelial cells [141, 142]. Therefore, DGBX decoction exhibits the potential to enhance the restoration of intestinal mucosal injury, alleviate local inflammation, and prevent carcinogenicity, as shown in Fig. 5.

### Balancing intestinal microbiota

Intestinal microbes and their metabolites influence not only the immune response but also the occurrence and development of CRC. Traditional Chinese medicines and their natural compounds are typically administered orally, inevitably interacting with the gut microbiota [234, 235]. Studies have demonstrated that astragalus polysaccharides effectively ameliorate colonic mucosal injury, restore immune homeostasis, and modulate the overall composition of the intestinal microbiota in mice with DSS-induced acute colitis. Furthermore, it normalizes the levels of *Firmicutes* and *Bacteroides* to their physiological states. In addition, astragalus polysaccharides after honey processing could increase the proportion of dominant bacteria such as *Lactobacillus* and *Bacteroides*, and significantly inhibit the upregulation of *Firmicutes* and *Verrucomicrobia*, thereby protecting the intestinal mucosa, affecting the diversity of microbiota, and alleviating the symptoms of colitis in mice. Honey-processed astragalus polysaccharides exhibited superior anti-inflammatory efficacy compared to astragalus polysaccharides in mice with colitis [100, 103]. The main components of *Astragalus mongholicus* Bunge-*Curcuma aromatica* Salisb. include calycosin, formononetin, and three astragalosides. The treatment effectively suppresses the proliferation of opportunistic pathogenic gut bacteria, such as *Shigella*, *Streptococcus*, and *Enterococcus,* while promoting the growth of beneficial probiotic gut microbiota including *Lactobacillus, Roseburia,* and *Mucispirillum.* At the same time, significant growth of colon cancer in tumor-bearing mice is inhibited and the intestinal barrier damage is repaired [236]. Interestingly, using human gut microbiota to mimic the gut environment, 4-vinylguaiacol (2-methoxy-4-vinylphenol), a metabolite of ferulic acid, exhibits stronger anti-cancer effects than ferulic acid on both chemo-resistant HT-29 and chemotherapy-sensitiveHCT116 cells. Therefore, oral ferulic acid provides a potential method for CRC treatment [237]. DGBX was found to partially restore the balance of intestinal microbiota destroyed by antibiotics and improve the abundance of intestinal microbiota by increasing the prevalence of *Bacteroides*, *Alistipes* and *Ruminiclostridium* [238]. Therefore, the utilization of DGBX decoction for gut microbiota modulation not only ameliorates colitis but also exerts inhibitory effects on colon cancer progression, thus exhibiting promising prospects in the management of IBD-associated CRC.

### Clinical research

A formulation developed from the DGBX decoction significantly ameliorates postoperative immunosuppression in cancer patients, sustainably bolsters immune function, and possesses anti-tumor properties, thereby promoting postoperative recovery [239]. In individuals sustaining severe abdominal trauma, there is a notable diminishment in cellular immunity. Clinical trials have evidenced that the administration of Astragalus injection as an adjuvant therapy is efficacious in the restoration of cellular immune function [240]. A Phase II clinical trial was conducted involving a cohort of healthy, naturally postmenopausal women. The study intervention involved the administration of escalating doses of oral DGBX decoction for a period of 12 weeks. Throughout the trial, physiological parameters and adverse events were closely monitored, with blood samples analyzed for a spectrum of health indicators. Notably, no significant alterations were observed in serum levels of total cholesterol, triglycerides, low-density lipoprotein cholesterol, or high-density lipoprotein cholesterol in either intra-group or inter-group comparative analyses. Further research is warranted to ascertain the potential therapeutic effects of DBT on blood lipid profiles in comparable populations [241]. Clinical studies also have demonstrated the efficacy of Astragalus extract TA-65 in ameliorating conditions associated with metabolic syndrome, including a significant elevation in high-density lipoprotein (HDL) cholesterol levels accompanied by a concurrent reduction in the low-density lipoprotein (LDL) to HDL cholesterol ratio, and a marked decrease in plasma TNF-α level [242, 243]. Some clinical trials of DGBX decoction and its main components are shown in Table 4.

**Table 4:** Some clinical trial of DGBX decoction and its main components

Study Title	Conditions	Intervention/treatment	Study design (enrollment/allocation/intervention model/masking)	Phase	Identifier
Study of Danggui Buxue Decoction in preventing neutropenia	Neutropenia Febrile neutropenia	Drug: DBD Drug: Epirubicin Drug: Cyclophosphamide Drug: Docetaxel	50 participants Randomized Parallel Assignment None (Open Label)	2	NCT02005783
Angelica sinensis for the treatment of hot flashes in men undergoing LHRH therapy for prostate cancer	Prostate cancer	Drug: Angelica Sinensis	44 participants Randomized Parallel Assignment Double (Participant, Investigator)	4	NCT00199485
To investigate the molecular mechanism of traditional Chinese medicine constitution using next-generation sequencing in nasopharyngeal carcinoma	Nasopharyngeal carcinoma	Drug: Danggui Buxue Tang Drug: Placebo	120 participants Randomized Parallel Assignment 120 subjects are divided into experimental group and control group (placebo) Triple (Participant, Care Provider, Investigator)	2	NCT03578575
Dose Finding Study of Danggui Buxue Tang (Herbal Formula) on the treatment of menopausal symptoms	Postmenopausal	Drug: DBT-Danggui Buxue Tang	60 participants Randomized Parallel Assignment Triple (Participant, Investigator, Outcomes Assessor)	2	NCT00420576
Effects of Danggui Buxue Tang on blood biochemical parameters in male recreational runners	Sports Anemia Inflammation Oxidative Stress Iron Deficiency Hemolysis Fatigue	Dietary Supplement: Danggui Buxue Tang Other: Placebo	39 participants Randomized Parallel Assignment Single (Participant)	Not Applicable	NCT02996786
Development of PHY606 as Adjunct therapy for anemia patients	Anemia Herba Interaction	Drug: Danggui BuxueTang (PHY606)	39 participants Randomized Parallel Assignment Double (Care Provider, Outcomes Assessor)	3	NCT04974073
The organ protection of astragalus in subjects with metabolic syndrome	Metabolic Sydrome	Drug: low dose Astragalus Drug: high dose Astagalus	210 participants Randomized Parallel Assignment Single (Participant)	3	NCT01847807
The integrated traditional chinese and western medicine treat early stage DKD	Diabetic Nephropathy Type 2	Drug: HuangQi Decoction Drug: HuangQi Decoction placebo	96 participants Randomized Parallel Assignment Double (Participant, Investigator)	1	NCT03681704
Astragalus membranaceus for brain edema induced by hemorrhagic stroke	Stroke Hemorrhagic Transformation Due to Acute Stroke	Drug: Chinese Herb Astragalus membranaceus Other: Placebo	80 participants Randomized Parallel Assignment Triple (Participant, Investigator, Outcomes Assessor)	2	NCT01428401
PG2 Treatment among stage II/III breast cancer patients receiving adjuvant chemotherapy	Cancer-related Fatigue Neutropenia, Malignant	Drug: Astragalus polysaccharides 500 mg Drug: Placebo Procedure: EC Chemotherapy	67 participants Randomized Parallel Assignment Quadruple (Participant, Care Provider, Investigator, Outcomes Assessor)	2	NCT03314805
PG2 treatment for improving fatigue among advanced cancer patients under standard palliative care	Cancer-related Fatigue	Drug: Astragalus Polysaccharides 500 mg Drug: Astragalus Polysaccharides 250 mg	323 participants Randomized Parallel Assignment Quadruple (Participant, Care Provider, Investigator, Outcomes Assessor)	4	NCT01720550
Effects of PG2 on fatigue-related symptom clusters	Cancer-related Fatigue	Drug: Astragalus Polysaccharides 500 mg Drug: Astragalus Polysaccharides 250 mg	6 participants Randomized Parallel Assignment Quadruple (Participant, Care Provider, Investigator, Outcomes Assessor)	Not Applicable	NCT02740959

## Conclusion

Immunometabolism, the intricate interplay between immune cell metabolism and immune function, has emerged as a promising field with potential therapeutic utility in various pathophysiological conditions. The anti-inflammatory and anti-cancer properties of AR and ASR within the traditional Chinese prescription DGBX decoction, prefigures its immunometabolism potential utility in the context of inflammation-cancer transformation, particularly in the setting of IBD-related CRC. It is evidenced by promoting intestinal mucosal repair and balancing intestinal microbiota. While the field of immunometabolism has made significant strides, it is important to acknowledge the limitations inherent in current research methodologies, such as the choice of experimental models, the fundamental biological differences between mice and humans, and clinical verification in the future. Further investigation into the therapeutic application of DGBX decoction for colorectal cancer is imperative, with a particular focus on elucidating its underlying mechanisms of immunometabolism modulation. Concurrently, it is crucial to implement stringent quality control measures and to standardize the production process of DGBX decoction to ensure its safety and reliability for clinical use.
